# The stream of experience when watching artistic movies. Dynamic aesthetic effects revealed by the Continuous Evaluation Procedure (CEP)

**DOI:** 10.3389/fpsyg.2015.00365

**Published:** 2015-03-31

**Authors:** Claudia Muth, Marius H. Raab, Claus-Christian Carbon

**Affiliations:** ^1^Department of General Psychology and Methodology, University of BambergBamberg, Germany; ^2^Bamberg Graduate School of Affective and Cognitive Sciences, University of BambergBamberg, Germany

**Keywords:** aesthetics, Aesthetic Aha, art, dynamic appreciation, indeterminacy, ambiguity

## Abstract

Research in perception and appreciation is often focused on snapshots, stills of experience. Static approaches allow for multidimensional assessment, but are unable to catch the crucial dynamics of affective and perceptual processes; for instance, aesthetic phenomena such as the “Aesthetic-Aha” (the increase in liking after the sudden detection of Gestalt), effects of expectation, or Berlyne's idea that “disorientation” with a “promise of success” elicits interest. We conducted empirical studies on indeterminate artistic movies depicting the evolution and metamorphosis of Gestalt and investigated (i) the effects of sudden perceptual insights on liking; that is, “Aesthetic Aha”-effects, (ii) the dynamics of interest before moments of insight, and (iii) the dynamics of complexity before and after moments of insight. Via the so-called Continuous Evaluation Procedure (CEP) enabling analogous evaluation in a continuous way, participants assessed the material on two aesthetic dimensions blockwise either in a gallery or a laboratory. The material's inherent dynamics were described via assessments of liking, interest, determinacy, and surprise along with a computational analysis on the variable complexity. We identified moments of insight as peaks in determinacy and surprise. Statistically significant changes in liking and interest demonstrated that: (i) insights increase liking, (ii) interest already increases 1500 ms before such moments of insight, supporting the idea that it is evoked by an expectation of understanding, and (iii) insights occur during increasing complexity. We propose a preliminary model of dynamics in liking and interest with regard to complexity and perceptual insight and discuss descriptions of participants' experiences of insight. Our results point to the importance of systematic analyses of dynamics in art perception and appreciation.

## Introduction

### An encounter with a salami made of wood

**Figure 1:**
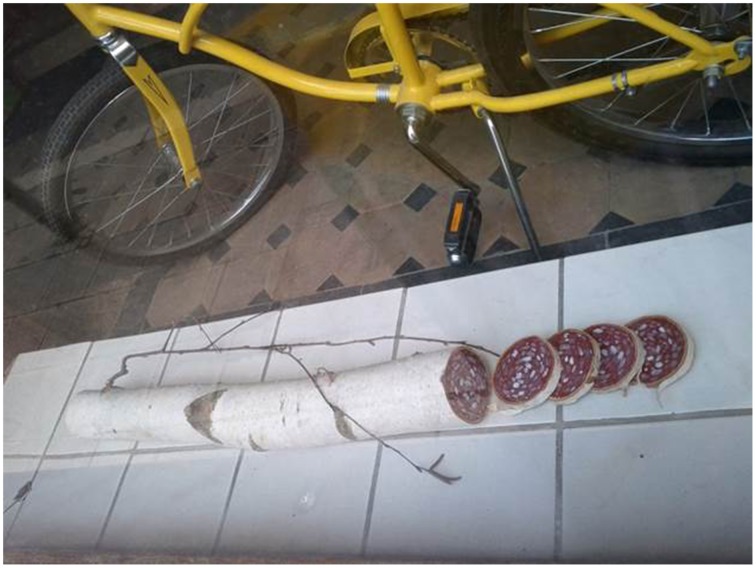
**Piece of wood, detected in a window in Nuremberg in 2013**. Photograph by Claudia Muth. Image courtesy of Claudia Muth.

If you had taken a walk along a calm side road in Nuremberg last summer, you might have encountered a strange object in a window (Figure 1). Surprised, you might have stopped there wondering why one would put a big piece of sausage—actually appearing to be Milanese salami—in a sunny window. On second glance, it might have appeared to you that this object is actually not a sausage but a piece of wood cut into pieces, with the natural white of the birch bark looking like salami casing, and sausage-like patterns painted on the cut edges. This insight might furthermore have made you wonder about the function of this place: is it a gallery? And finally, despite being amused, you might have even felt a bit fooled as the illusion was obviously deliberately intended for pedestrians and made you an unwitting part of an artistic project. This little episode shows that we are guided by expectations, continuously forming predictions about the world, and that we are easily irritated when they are not met. The underlying mechanism is described in the cognitive sciences as “predictive coding”; a theory deeply rooted in the concept of perception as knowledge-driven inference proposed by psycho–physiologist Von Helmholtz (1866). Within this conceptual framework it is stated that instead of a bottom-up accumulation of information we engage constantly in matching sensory inputs with predictions created on the basis of prior experiences (for a recent critical examination of this account see Clark (2013). Artists widely make use of mismatches between predictions and actual sensory cues by providing deviations from beholders' expectations or perceptual habits. According to the *tentative prediction error account of visual art* (Van de Cruys and Wagemans, 2011) these mismatches motivate the perceiver to engage in the rewarding resolution of the prediction error.

### Physical and semantical dynamics in perception

Two main conclusions might follow on from this:

- Our perceptual impressions of an object and its context are in permanent flux as we move or as the object moves or transforms itself: the (perceived) world is not static but permanently *physically changing*.
- Meaning evolves out of interaction with our environment and is not obvious and inherent *per se*: the (perceived) world is continuously re-evaluated and thus not determinate but *semantically changing*. This means that, even if something remains physically constant, our attribution of meaning is permanently updated by cognitive and affective processes. Perception is always based on psychological processing which is highly interactive and dependent of expectations, predictions, and activation of semantic networks (see Carbon, 2014).

In the realm of this research project, we examine the dynamics of such changes. These are qualities or specific patterns of changes, respectively. Dynamics can emerge out of physical changes (e.g., the typical dynamics of an explosion), semantic changes (e.g., the dynamics of a sudden shift in valence when learning about the financial value of a vase which you are holding in your hands), or their interaction (e.g., the dynamics of a perceptual Aha insight when finding Wally in a crowd after scanning the scene via eye movements). Semantical dynamics become specifically evident in the perception of objects that offer multiple meanings and afford elaboration: the “wooden sausage” (see Figure 1), for instance, induces dynamics specific to the phenomenon of bistable ambiguity as we switch from one determinate interpretation (sausage) to the other (wood) and eventually back again (see the psychological concept of ambiguity by Zeki, 2004, as well as the conceptualization of multistability as defined by Kubovy, 1994).

**Figure 2:**
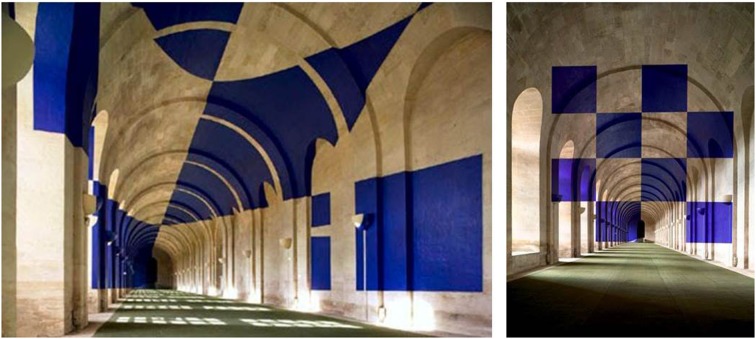
**Varini (2006)**. Huit carrés. Versailles: Orangerie du Chateau de Versailles. Image courtesy of Felice Varini.

**Figure 3:**
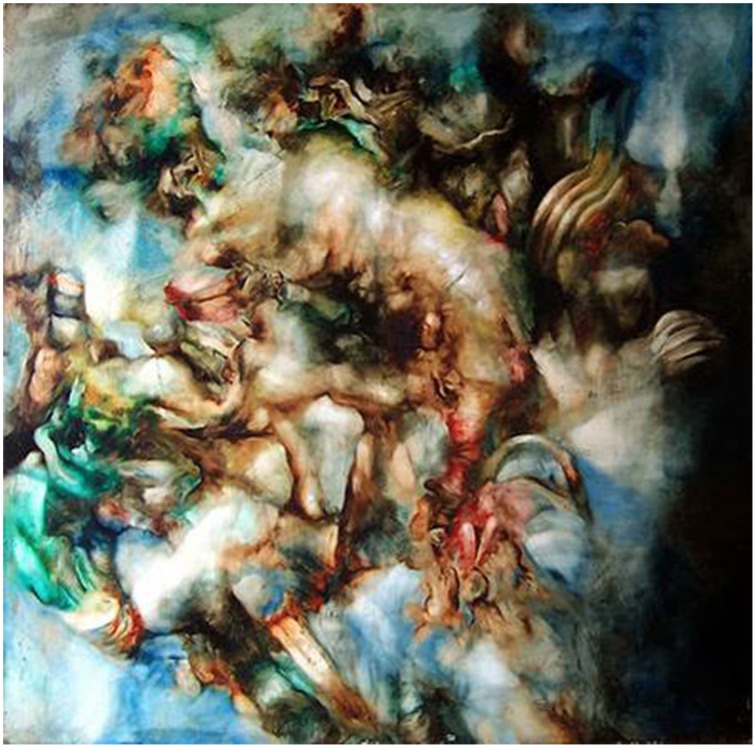
***Succulus*, a painting made by Pepperell (2005)**. Image courtesy of Robert Pepperell.

Evidently, physical and semantical dynamics are linked as we can imagine physical changes inducing semantical changes. Varini's (2006) “Huit carrés,” for instance, plays with perceptual changes induced by different viewpoints of the object. This way they surprisingly reveal appearances of Gestalt from a specific physical viewpoint (see Figure 2). His works thus fuse physical with semantical dynamics. By concealing an identifiable pattern his work exemplifies the art theoretical definition of “hidden images” by Gamboni (2002), challenging the recipient to search (cognitively, but also by moving around or in front of the artwork) for an object that is actually present in the image but cannot be perceived too easily. A special class of semantical dynamics is found in “potential images” which—in contrast to hidden and ambiguous images—are fully indeterminate. They do not provide any recognizable object but are evocative of something we might know (for “potential images” see Gamboni, 2002; for indeterminacy see Pepperell, 2006). The example in Figure 3 stimulates all kinds of association and motivates intense exploration without resolving into certain, determinate identification (in contrast to ambiguity, which offers several certainties with the same probability; see definition by Zeki, 2004). As the art historian Gombrich (1960) proposed for the perception of Cubist artworks—offering a wide range of indeterminacy—here also the visual search continues after cues have been detected. Indeterminacy is thus a suitable phenomenon for studying highly dynamic experiences in regard to the attribution of meaning.

### Dynamics in liking

It is quite clear that not only are dynamics to be found in the perceptual process itself and the processing of semantics, but aesthetic appreciation is obviously changeable, too. This has been an issue of empirical studies in the domain of psychological aesthetics, though there remains to be a systematic examination of this process. For instance, appreciation was found to increase with unreinforced visual presentation of a stimulus (see mere-exposure effect by Zajonc, 1968) limited by the factor of increasing boredom (Bornstein, 1989) or fatigue (Carbon, 2011). While here, increasing familiarity seems to be a crucial factor, the depth of elaboration played a role in a study by Carbon and Leder (2005): innovative car designs were liked more after elaboration of the material via the so-called Repeated Evaluation Technique (RET; cf. Faerber et al., 2010). A further example of empirical evidence for dynamics relevant to appreciation is demonstrated by the phenomenon of the “Aesthetic-Aha” (Muth and Carbon, 2013), stating that the recognition of a Gestalt within two-tone-images yields a sudden increase in liking. Positive effects of the subjective strength of perceptual and cognitive insights on appreciation were also reported in the domain of art perception (Muth et al., 2015).

The mere exposure is referred to in so called fluency theories: higher familiarity by repetition leads to an increase in processing fluency which is marked by positive affect (e.g., Reber et al., 2004). The elaboration of the car interior designs—in contrast—comprised an active engagement which probably induced changes in the perception of the material's features and qualities. Here, as well as in regard to the “Aesthetic Aha” effect, dynamics in appreciation are linked to dynamics in semantics elicited by deep elaboration or increases in determinacy (Aha!), respectively. While fluency might actually also play a role in such semantical dynamics, it might be crucial that the material itself is difficult or complex enough to induce an increase in certainty in the first place for the positive effect of an “Aesthetic Aha” to occur: while in the rationale of fluency accounts we prefer the most predictable stimulus, Van de Cruys and Wagemans (2011) point to pleasure from the “transition from a state of uncertainty to a state of increased predictability” (p. 1035). The sudden revelation of a Gestalt within an indeterminate picture thus might be a rewarding resolution of indeterminacy.

Still, as the philosopher Gadamer (1960/2002) points out “There is no absolute progress and no final exhaustion of what lies in a work of art” (p. 100). Therefore, we would like to add that even if increases in predictability or certainty, respectively, are pleasurable this does not have to mean that we derive pleasure solely from arriving at a fully determinate interpretation of an artwork (if possible at all). Here, the semantical dynamics might not equal the pattern of a unidirectional progress in regard to uncertainty reduction, but instead might consist of an alternation between indeterminate phases, moments of insight, or even an endless loop among determinate patterns within ambiguous objects. Partial insights into the semantic structures of an artwork (e.g., discovering the topic of the depicted scene while still being puzzled by the choice of stylistic means) might evoke pleasure without providing “a solution” to the “problem” posed by the work (see Muth et al., 2015). Such insights might happen several times during the elaboration of an artwork and might be an important factor why many pieces of art keep the beholders' interest in them alive.

### Dynamics in interest

Aside from the actual changes in meaning attribution, the expectation of insight might also be relevant to one crucial dimension of appreciation: interest. Berlyne (1971) assumed “disorientation” with a “promise of success” to elicit interest, while Silvia (2005) proposed a combination of appraisals for interest—one being the challenging features of an object, and the other being one's ability to cope with these challenges through understanding. Thus, even before a sudden increase in determinacy, the interestingness of an object might increase. For indeterminate objects like the one depicted in Figure 3 this might be particularly relevant: while not providing the experience of total determinacy, interest might arise due to the ongoing “promise,” the permanent unresolved “potential” of determinacy.

### Aims and hypotheses

We aim at providing a more elaborate picture of the interplay of physical and semantical dynamics with dynamics of appreciation by assessing continuous evaluations of movies in regard to the dimensions of complexity (physical dynamics), (in)determinacy and surprise (semantical dynamics), as well as liking and interest (dynamics of appreciation). We examined (i) the effects of sudden perceptual insights (increases in surprise and determinacy) on liking; that is, “Aesthetic Aha”-effects, (ii) the dynamics of interest before moments of insight, and (iii) the dynamics of complexity before and after moments of insight. Based upon the reported findings of the “Aesthetic Aha” effect (Muth and Carbon, 2013) as well as the theoretical account of reward by uncertainty reduction (Van de Cruys and Wagemans, 2011) we predict (i) an increase in liking at moments of insight, while (ii) interest might already increase before moments of insight due to the anticipation of success (Berlyne, 1971; Silvia, 2005). For a moment of insight to happen, (iii) a certain level of complexity might be necessary.

## Materials and methods

### Participants

Sixty participants took part in the experiment on a voluntary basis (28 participants in a gallery setting, mean_age_ = 38.1 years; range_age_ = 18–85 years and 32 participants in a laboratory, mean_age_ = 20.7 years; range_age_ = 18–24 years). A *Snellen* eye chart test and a test with a subset of the *Ishihara* color cards assured that all of them had normal or corrected-to-normal visual acuity and normal color vision. The participants were naïve to the purpose of the study.

### Apparatus and stimuli

**Figure 4:**
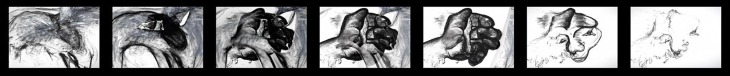
**Exemplary frames of the stop-motion movie *Konstrukte* by Claudia Muth (from the year 2009)**. Image courtesy of Claudia Muth.

As material we employed “Konstrukte”—a movie (07:18 min.) by Claudia Muth (from the year 2009) which was created in an artistic context, originally not intended to be stimulus material. It depicts the evolution and metamorphosis of Gestalt (see Figure 4 and Supplementary Material A) and the changes in determinacy are well suited for the study of physical dynamics (changes among subsequent film stills and their complexity) as well as semantical dynamics (emergence, disappearance, and metamorphosis of Gestalt). The artist used an intuitive drawing technique which allows for the development of Gestalt out of arbitrarily set lines to slowly reveal order in a seemingly diffuse picture and photographed the drawings (charcoal and acrylic paint) in various stages differing only slightly in detail (stop-motion technique). This way the process of drawing as well as the artist's associations can be retraced. It is suitable as material for this study because it consists of a dynamic variation of (in)determinacy; the associations of the perceiver might be forced or destroyed and insights induced. While these preconditions might be met by static indeterminate pictures as well, utilizing a material which is dynamic in itself allows for the definition of a wide range of physical as well as semantical dynamics and their temporal comparison within and between participants' evaluations. The movie was presented on a LG W2220P screen with a 22-inch screen size and a resolution of 1680 × 1050 pixels.

**Figure 5:**
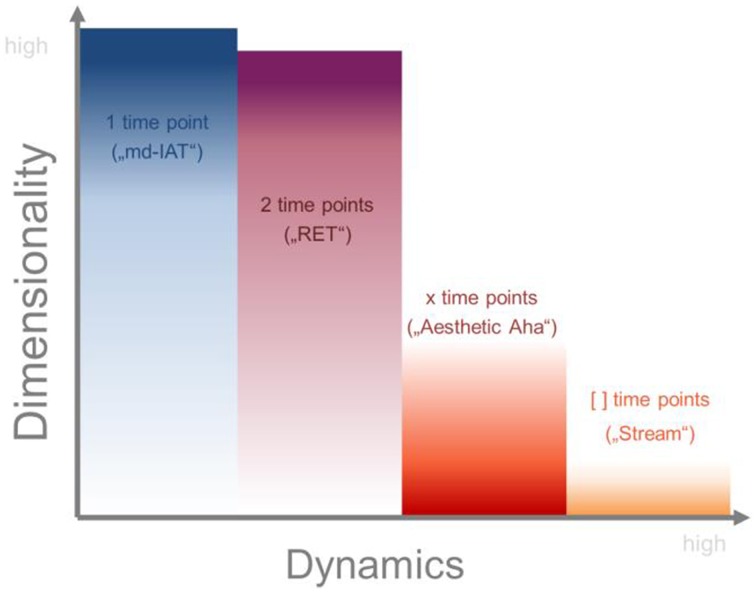
**Typical dimensionality and dynamics of assessments with different temporal resolution**. Adapted from Raab et al. (2013).

As an input device we developed an apparatus that is able to capture assessments in a very time-accurate way. Research in visual perception science is often based on snapshots, moments, or stills of experience. Static approaches allow for differentiated and deep assessment, but are hardly able to catch the dynamics of affective and perceptual processes which—as we exemplified above—are actually crucial for certain aesthetic effects. Figure 5 shows a broad overview on different kinds of assessment ranging from low (data measured at one time point) to high (interval of data) temporal resolution. One-time point-measurements allow for deep multidimensional assessments (e.g., the multidimensional extension of the IAT, called md-IAT, see Gattol et al., 2011). The more time points are introduced, the higher the resolution of captured dynamics; but also at the same time, the fewer the dimensions which can be included due to time constraints and problematic effects of order and fatigue. Two-time point measurements like the RET (Repeated Evaluation Technique, measuring appreciation before and after elaboration, see Carbon and Leder, 2005) can still include various dimensions but capture only coarse changes between distinct points of assessment (mostly only up to 4 such time points, see Carbon et al., 2013). The “Aesthetic Aha”-effect was revealed by a finer-grained picture of changes in face clarity and changes in the liking of a picture by introducing 12 time points of measurement (Muth and Carbon, 2013). One-dimensional but temporally highly resolved assessments are achieved by, e.g., pupillometry, electro-dermal activity, or electromyography. Nevertheless, there remains a gap between such measurements and the respective qualifications of the affective states in such instances: electro-dermal activity, for instance, might be an interesting indicator of affective strength, but not of affective valence (Carbon et al., 2008).

To provide a highly dynamic assessment of evaluations of the artistic films we used a method we would like to call Continuous Evaluation Procedure (CEP; developed by the research network *Ergonomics, Psychological* Æsthetics, and Gestaltung, EPÆG), realized by employing a do-it-yourself built slider box as the input device. The CEP provides a more fine-grained picture of aesthetic assessments, and therefore also for analyzing effects like the “Aesthetic Aha” (Muth and Carbon, 2013). The system comprises a standard lever which is typically used for audio equipment (100 mm movement range, 10 kΩ linear characteristics), mounted on wooden housing. Inside the box, an ATMEGA microprocessor continuously measured the lever's resistance, mapped the resistance to a value between 80 and 1024 (with 80 indicating the lowest and 1024 the highest lever position; referred to as “strength” in the following and transposed to a scale ranging from 0 to 1 in the figures for better readability) and sent the value via an FTDI serial-to-USB converter to the connected PC. The current slider position was stored in the box as a numerical value, and updated constantly and virtually without time delay by an ATMEGA processor. Upon each new movie frame, the current value (that is, slider position) was requested via the Serial-to-USB interface. For our setup, this meant slider positions for a movie running at 30 fps could be recorded without introducing a time lag. The video presentation was realized via the Processing Library for Visual Arts and Design (Fry and Reas, 2014) and the GStreamer library (Open-Source, 2014), in which for each movie frame the current slider position was retrieved and stored. To achieve multidimensionality we used the CEP repeatedly for all key variables.

### Procedure

**Figure 6:**
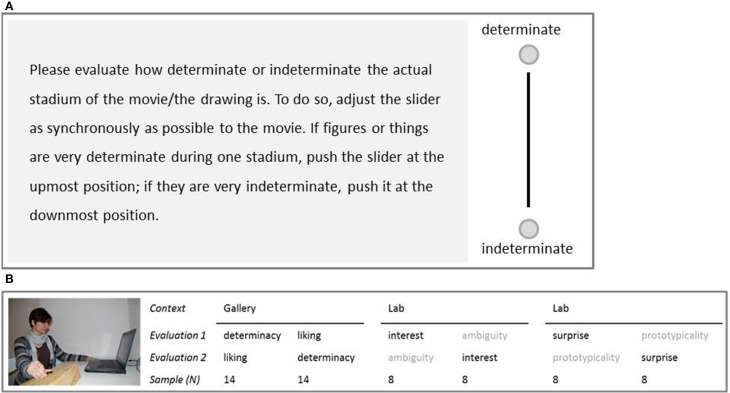
**(A)** PExemplary instruction for key variable “determinacy”; **(B)** Experimental setting and counterbalanced procedure (variables in gray color are not integrated in further analyses; variables in black are key variables). Image courtesy of Claudia Muth.

Every participant watched the movie twice and evaluated it continuously on one dimension each time via the CEP. The instructions were given together with a graphical representation of the slider and the two poles of the according key dimension (see Figure 6A). Afterwards the participants were asked to push the slider up and down to get a feeling for the usability of the apparatus. One group then evaluated the key dimensions of liking and determinacy in two subsequent trials in an art gallery (“Griesbadgalerie” in Ulm, Germany; in a room separate from the exhibition, see Supplementary Material B). To minimize order effects, the order of the two dimensions was counterbalanced (for a visualization of the setting and the rationale of the counterbalanced design see Figure 6B). These dimensions were complemented by further testing sessions with other groups of people on the dimensions of surprise at an experimental laboratory at the University of Bamberg, Germany to be able to define insight moments as a combination of determinacy and surprise. Furthermore, we included interest as a second dimension of aesthetic appreciation by additional assessment in the same lab setting. We decided to follow this strategy as we were mainly interested in liking and determinacy and aimed to test these variables under the ecological condition of a gallery context. But to test in a gallery also means to limit the experimental approach: precisely, when testing in a gallery, the number of volunteering gallery visitors is limited, and testing runs the risk of disturbing the experience of other visitors. This made us develop the design of capturing the two key variables of aesthetic experience in the gallery and the additional variables in the laboratory (all variables were asked for in an order-balanced way). We stuck to this one-person-two-dimensions design for the gallery testing for other aesthetic factors, too, in order to keep the design consistent. After the second evaluation phase, participants filled out a questionnaire to “describe in a few own words how it felt to suddenly recognize something clearly.”

## Analysis

**Figure 7:**
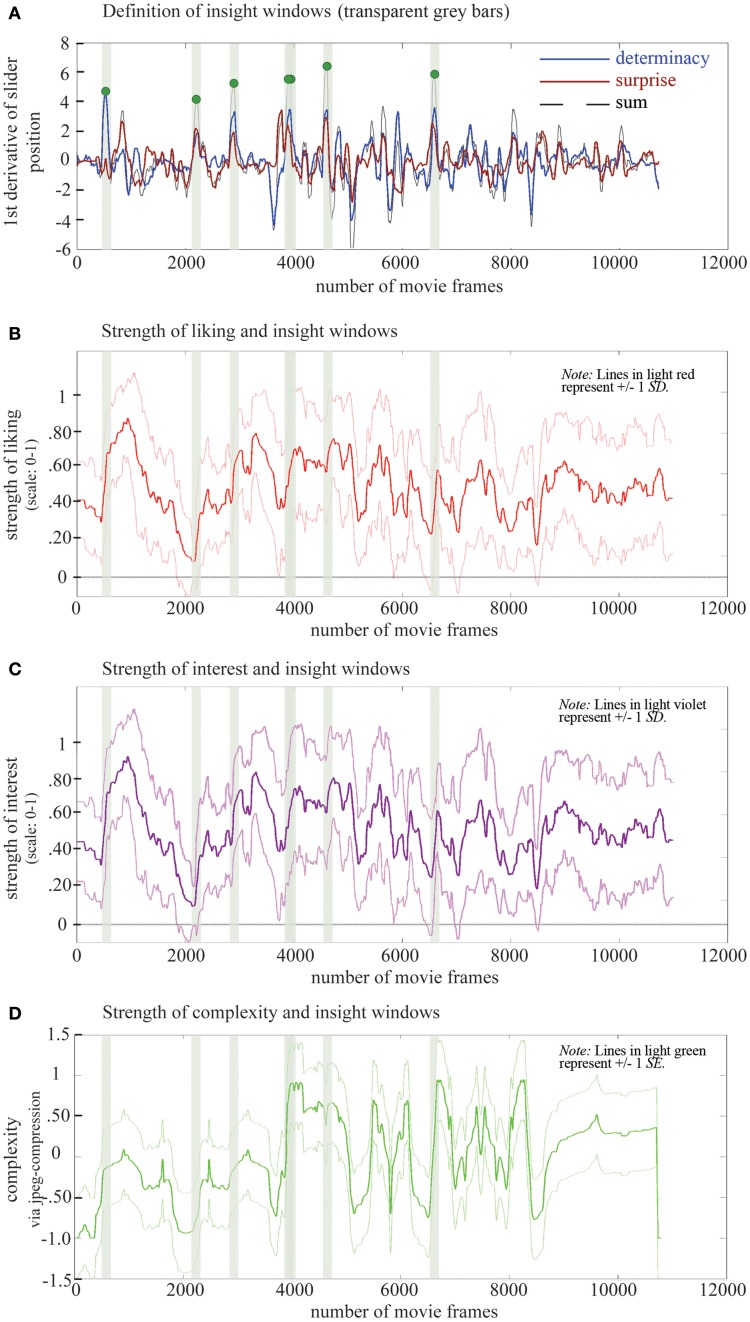
**(A)** Definition of insight windows: 1st derivative of strength of determinacy and surprise (indicated by the lever position), their sum and the moments of insight, marked by green dots. **(B–D)** Dynamics in liking, interest, and complexity in relation to insight windows. Note that 2000 movie frames correspond to about 67 s.

To define moments of insight, we described the movie's dynamics in four dimensions: three of them based on empirical data for determinacy, surprise, and interest along with data on complexity based on an automatized analysis (size of each frame after jpeg-compression, see Marin and Leder, 2013). We then identified moments of insight by (a) determining local maxima in the first derivative of ratings (averaged over participants) on determinacy and surprise, respectively and (b) identifying points in time where both derivatives reach common peaks. This follows the definition of insight as a sudden (strong surprise) and clear (high determinacy) solution to a problem (as proposed in the 1930s by Gestalt psychologists and redefined recently, e.g., by Bowden et al., 2005). We selected those peaks in which (a) the highest sum of both variables is achieved and (b) the sum of both variables contributes to the peak resulting in seven moments of insight (see Figure 7A and Supplementary Material C). While these peaks mark the points of biggest change in their respective dimension, we assume that the psychologically relevant event—the insight—had occurred shortly before the rapid increase detected by the CEP. By visual inspection of the yielding data curves, we determined this insight point (when the lever movement leading to the rapid change began to show in the data) to be 45 frames prior to the peak. So for any peak showing at *t* = *x* (*x* being the movie frame), the insight was located at *t* = *x*−45.

**Figure 8:**
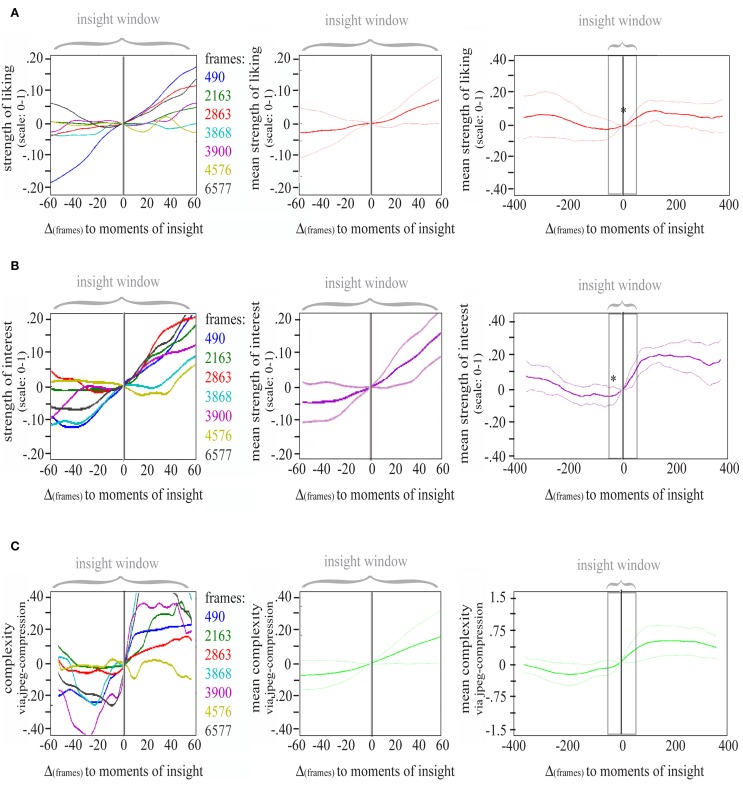
**(A)** LIKING Left panel: seven phase-shifted liking evaluations at the insight windows. Middle panel: averaged liking evaluations at the insight windows. Right panel: insight window and adjacent data windows with the only significant change being marked by an asterisk. Note: lines in light red represent the standard deviation, 2000 movie frames correspond to about 67 s. **(B)** INTEREST Left panel: seven phase-shifted interest evaluations at the insight windows. Middle panel: averaged interest evaluations at the insight windows. Right panel: insight window and adjacent data windows with the only significant change being marked by an asterisk. Note: lines in light violet represent the standard deviation, 2000 movie frames correspond to about 67 s. **(C)** COMPLEXITY Left panel: seven phase-shifted complexity evaluations (size of each frame after jpeg-compression, see Marin and Leder, 2013) at the insight windows. Middle panel: averaged complexity evaluations at the insight windows. Right panel: insight window and adjacent data windows. Note: lines in light green represent the standard error, 2000 movie frames correspond to about 67 s.

**Figure 9:**
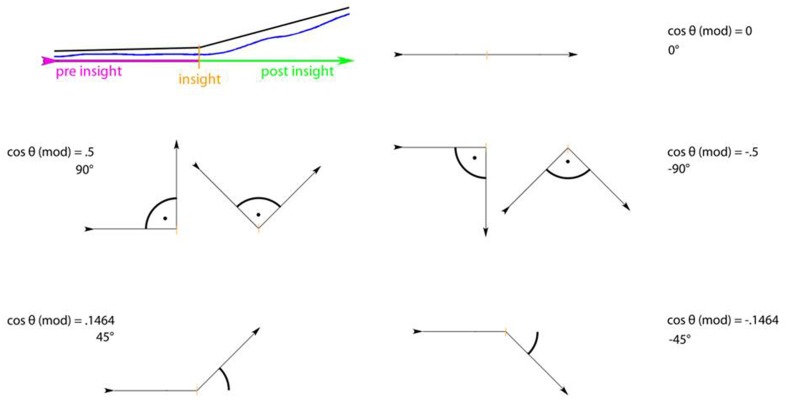
**The modified cosine theta measure (Θ) to capture dissimilarity between pre- and post-insight**. The data section before the insight as well as after the insight was approximated with a line each (top left). Between these two vectors, the angle was determined. Two vectors with exactly the same direction (i.e., with no difference in direction pre- and post-insight) result in an angle of 0° between vectors and thus in a cosine theta measure of 0. A directional change upwards (second vector pointing higher than the first one) will result in a positive theta (e.g., 0.5 for a 90° upward angle between vectors); a directional change downward in a negative theta value (e.g., −0.5 for a 90° downward angle).

To reveal dynamics in appreciation with regard to an insight moment, we selected seven data intervals (*insight windows*) containing liking ratings ranging from 60 frames prior to each insight moment to 60 frames (which equals 4 s overall) after that insight moment and selected the according intervals of liking data (see Figure 7). We then phase-shifted all seven time windows around the insights to obtain one single *insight window* in which each insight moment is marked by frame “0” to be able to compare all changes in liking in relation to insight (see Figure 8A left). We then averaged data (see Figure 8 middle) and used a modified cosine value of the angle between the slope describing data before (frame “–60” to frame “0”) and the slope describing data after the insight moment (frame “0” to frame “60”; see Figure 9).

The cosine measure is a common metric in the field of information retrieval for determining similarity between two vectors (see Singhal, 2001). It results in “1” when two (*n*-dimensional) vectors point in exactly the same direction; it is “0” for orthogonal vectors; and “–1” for opposing vectors. Here, we rotated and re-assembled the measure in such a way that it captures the dissimilarity between two vectors. It is “0” when both vectors (pre- and post-insight) have the same direction; it approaches “1” when the post-vector marks an increase compared to the pre-vector (where “0.5” would be an angle of 90°); and it approaches “–0.5” when the post-vector marks a decrease (Figure 9).

The cosine value obtained for the *insight window* thus describes changes in the corresponding variable, e.g., liking, at the insight moment. To test if this change is significantly different from the general dynamics of liking evaluations, we compared the seven cosine values at the moment of insight to those of 1000 randomly picked data intervals (*non-insight windows*) and conducted a *t*-test to check if the cosine values at the *insight windows* are distinguishable from the random sample.

## Results

### Effects of sudden perceptual insight (“Aesthetic Aha”)

**Figure 10:**
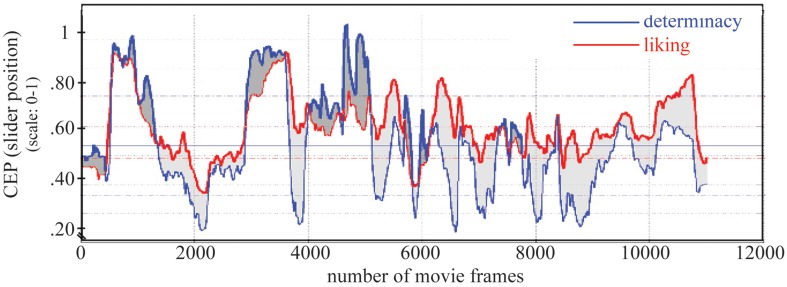
**Strength of determinacy and liking**. The difference of both variables is indicated by gray colored areas.

**Figure 11:**
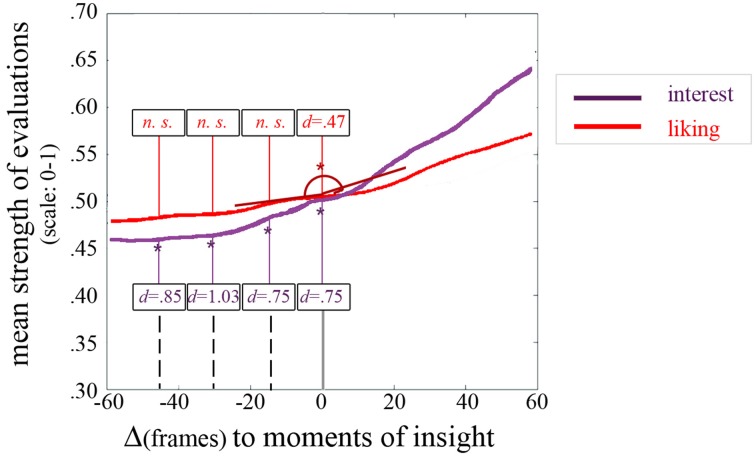
***Insight window* revealing the dynamics of liking and interest in relation to moments of insight**. An exemplary angle from which we derived the cosine value that we compared to a random set of 1000 cosine values of other changes is depicted for liking at the moment of insight. Numbers signify the strength of the effect via Cohen's *d* for significant changes at the insight moment [Δ(frames) = 0] as well as prior to the moments of insight [at Δ(frames) = –15, Δ(frames) = –30, and Δ(frames) = –45]. Note: changes which are significant at an α-level of 5% are marked by an asterisk.

A two-sided one sample *t*-test revealed that the increase in liking during an *insight window*, meaning after an insight moment, was significantly higher than other changes in liking during the evaluation of the movie, *t*_(1005)_ = 2.33, *p* = 0.02, Cohen's *d* = 0.47 (for a visual comparison to changes in adjacent windows see right panel in Figure 8). Changes before that time point were not significant (for a differentiated visualization of these results see Figure 11). Furthermore, we found a strong correlation between determinacy and liking (*r* = 0.663, *p* < 0.001; see Figure 10).

### Dynamics of interest before moments of insight

Analogous to the analysis of changes in liking in relation to insight moments, we conducted analyses on the changes in interest (with the modified cosine measure) and revealed that interest already increased 45 frames/1500 ms before the moment of insight, *t*_(1005)_ = 2.53, *p* = 0.01, Cohen's *d* = 0.85 (interestingly, even stronger than at the insight moment itself; see also Figure 8B, right panel and Figure 11). The increase was strongest 30 frames/1000 ms before the moment of insight, *t*_(1005)_ = 3.78, *p* < 0.001, Cohen's *d* = 1.03. Weaker, but still large effect sizes were found 15 frames/500 ms before the insight point *t*_(1005)_ = 3.18, *p* = 0.002, Cohen's *d* = 0.75, and at the insight moment, *t*_(1005)_ = 2.64, *p* = 0.01, Cohen's *d* = 0.75. We furthermore found a strong correlation between determinacy and interest (*r* = 0.767, *p* < 0.001).

### Dynamics in complexity before and after moments of insight

Plotting a phase-shifted *insight window* of complexity revealed that insights happened during an increase in complexity (Figure 8C).

## Discussion

We examined (i) effects of sudden perceptual insights (increases in surprise and determinacy) on liking; that is, “Aesthetic Aha”-effects, (ii) dynamics of interest before moments of insight, and (iii) dynamics of complexity before and after moments of insight while watching an artistic movie. Our findings showed that the analysis of a continuous stream of data can reveal dynamic relationships between an artwork's physical and semantical dynamics and appreciation. Such analyses demonstrate that aesthetic experiences unfold in a dynamic way: (i) insights indeed elicited an “Aesthetic Aha,” an increase in liking (as in Muth and Carbon, 2013). (ii) The rise in interest before an insight supports Berlyne's (1971) as well as Silvia's (2005) ideas that interest is evoked by (the appraisal of) challenge and coping potential—the expectation of success. (iii) Furthermore, it seems that for an insight to occur, stimuli have to possess a certain complexity. In dynamic terms this means that only phases in the movie in which complexity increases have the potential to lead to insightful moments—which again evoke an increase in liking. This is in accordance with the idea proposed by Van de Cruys and Wagemans (2011) that it is not predictability itself but the reduction of uncertainty induced by prediction-errors that might bring pleasure. There are two directions of interpretation of these three results: either (a) the Aha-insight benefits from, or is more probable because of, an orienting reaction and high interest due to an increase in complexity or (b) interest arises due to “affective forecasting” (“people's predictions about their future feelings,” see (Wilson and Gilbert, 2003); Wilson and Gilbert, p. 346) of this Aha-insight. In addition, the experience of an Aha-insight itself might stimulate the orienting reaction and deeper elaboration of subsequent phases as is exemplified by descriptions of participants, for instance the following: “proud of myself, motivated to continue watching (maybe one detects something else/more),” “I concentrated on the monitor and every recognition of a “picture” pleased me and incited me to search even more “intensely” (with more concentration) for more “pictures.”

Such links between perception, affection, and appreciation suggest that systematic analyses of dynamics in art perception are crucial for an understanding of the unfolding of meaning as well as the experience of art in general. Still, people's descriptions of their experiences of an Aha-insight show that there might be additional mechanisms involved in the links between complexity, insight, and appreciation. To stimulate further discussion, we exemplify three noticeable aspects which were frequently present in the *post*-*hoc* descriptions of the participants' own experiences of Aha-insight moments in Table 1. One aspect regards descriptions of familiarity or related concepts such as “control,” “relatedness,” “success,” or “relieve” which might point to the appraisal of coping potential (see 1st column in Table 1). Another less frequently mentioned aspect is the relationship between expectation and resulting recognition, and their effect on appreciation. Two different possibilities were present in the descriptions: prediction confirmation as well as prediction error were experienced as pleasurable (see 2nd column in Table 1). A further aspect regards the distinction between process-focused and result-focused elaboration, when participants mentioned “transitions” between Gestalt perception or reported having enjoyed the process itself vs. when they described the search for recognizable Gestalt (see 3rd column in Table 1).

**Table 1:** **Examples of participants' descriptions of their experiences of Aha Insight moments, translated by the authors**.

**Coping**	**Prediction confirmation vs. prediction error**	**Process-focus vs. result-focus**
“A feeling of ‘familiarity”’	“[…] can be negative then, if the picture (where one recognizes something) does not conform to the expectation.”	“Pleasing and partially surprising. The development into a new form, the process of transition is however more interesting than the final result (in this case the clearness).”
“Insight, relief, higher relatedness to the picture”	“Pleasing because assumption got confirmed, then a little bit disappointed that it was what one suspected.”	“[…] also it is fascinating to recognize something new, especially if you consider what it evolved out of.”
“Relieved and maybe a bit proud”	“[…] at the same time also happy or less happy about what I saw. Sometimes also disappointed, that something other than what I thought would come became recognizable.”	“It was comfortable to detect things and to see them evolving.”
“Certain experience of success, positive feeling”	“[…] with the structure getting clearer and the result more foreseeable it got more ‘boring’.”	“I was excited by the change.”
“Additionally, the recognizable contours gave me a feeling of security or also ‘control’.”	“Partially it is also frustrating, if you have waited for the drawing to develop but could not recognize or classify anything.”	“It was interesting to recognize something out of ‘nothing.’ To identify something suddenly felt clearly better than the transitions among the different motives.”

The relationship between the prediction of a Gestalt and actual recognition of a Gestalt seems to have a very different effect on appreciation for different individuals: whereas some participants described a confirmation to be pleasurable, others clearly preferred surprising transitions within a movie. This difference might be very important in the domain of art perception as it seems to imply that the valence of any experience of new insights is mediated by perceptual and cognitive habits. All of these factors are potentially different in gallery visitors compared with volunteers, mostly art-naïve students, typically tested in laboratory conditions. Another speculation concerns the mode of perception: we might focus on the process of physical and semantical transformations or on the resulting recognizable Gestalt, respectively. It would be highly interesting to systematically and explicitly investigate in future studies how the activation of these modes of processing is related to personality and context factors.

The assessment of dynamics in perception provides insights not only in regard to continuous changes and dynamic relationships but also enables us to reveal effects of expectation. We compared the two groups of participants evaluating the surprise induced by the different phases in the movie either during its first or its second presentation. The pattern of the resulting data implied that it makes a difference if an observer expects the development of Gestalt or indeterminacy due to previous exposure or if predictions are formed by the available information only. As these differences neither seem to be systematic nor easily describable, e.g., by a shift of peaks to earlier phases, this point is left for following studies with a concise focus on effects of expectation.

**Figure 12:**
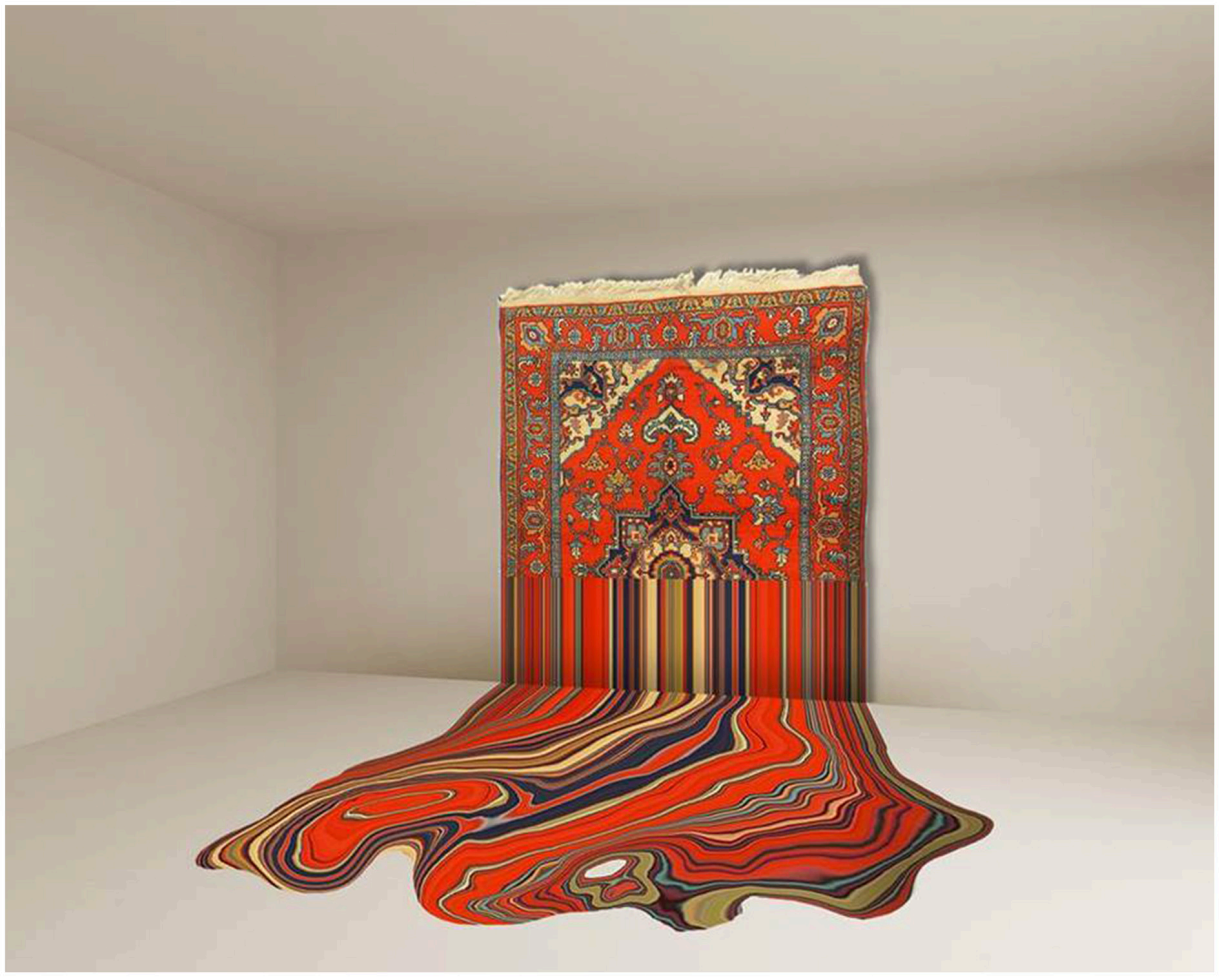
***Liquid* by Faig Ahmed in the year 2014 [Handwoven rug made of wool]**. Image courtesy of Cuadro Gallery, United Arab Emirates.

Art is not only an ideal medium through which physical and semantical dynamics in experience can be studied: while artworks might be insightful for perception science as they do not represent things as they are but as they are perceived (Fiedler, 1971), many artworks also do explicitly refer to and reflect on these dynamics. Some of Paul Cézanne's works hinder determinate interpretation and instead provide potential shapes of things. Gamboni (2002, p. 116) thus states that Cézanne leaves images “in perceptual formation and makes the spectator conscious of the interpretative process in which he is engaged and which will never be conclusive.” Also Majetschak (2003) related Cézanne's works to the constructive activity of perception and called them consequently a “birthplace of visibility” (translated from “Geburtsort von Sichtbarkeit,” p. 324). The dynamics of potential images (Gamboni, 2002) are also found in Cubist artworks by, e.g., Picasso and Braque which never provide full determinacy but contradictory cues which evoke an ongoing search for Gestalt (Gombrich, 1960). Furthermore, a Cubist artwork might allow for the retracing of part of the artist's dynamic elaboration of the original object: instead of a fixed spatial relation between painter and object, Cubist artists applied a “mobile perspective” (e.g., Metzinger, 1910), an “analytic description” of the object, or a “synthesis” of various viewpoints (translated from Kahnweiler, 1971, p. 69). This simultaneity of spatial dynamics within one picture might let us simulate an actual visual exploration which integrates several fragmented “semi-worlds” (Churchland et al., 1994)—inhomogeneous of detail and colorization—over time. Cubist artworks might thus be good examples of two kinds of semantical dynamics induced by the simultaneity of both potential identifiable forms and perspectives. Examples of the capturing of dynamics by artworks often regard the illusory layer of the artwork (the depicted objects or sceneries)—not the material layer (canvas and color). But semantical dynamics can also evolve in regard to the material itself. The carpets made by Faig Ahmed (Figure 12) are valuable examples of semantical dynamics due to switches between interpretations of the material (paint vs. carpet) along with other semantical levels like the historical, social, and cultural dimensions of traditional carpet weaving. Contemporary “Relational art” (Bourriaud, 2002) expands the dynamic relationship between artwork and perceiver even more as it points to or includes the social context of behavioral interactions. The Munich based group *Die Urbanauten*, for instance, organizes “Swarm-Happenings” in which people are instructed to fulfill urban interventions—actions in public space like having a picnic on a bridge—to reflect on and inspire discussions about how public space is used and the set of norms which underlie our behavioral variety. Works of art thus neither have to be exhibited in a well-defined artistic context (gallery, museum) and contemplated by one observer alone, nor is the authorship and evaluation of these works independent of the social context. This viewpoint opens up a variety of possible dynamic levels that art perception comprises; the presented investigation is a first step into this wide and thrilling field of art and research.

## Outlook: a preliminary model for physical and semantical dynamics in liking and interest

**Figure 13:**
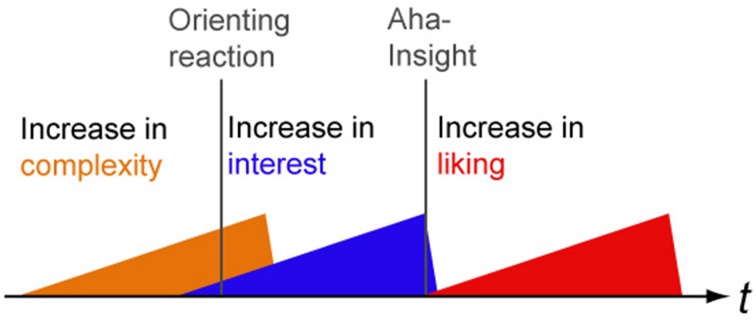
**A preliminary model of physical and semantical dynamics in interest and liking**. An increase in complexity elicits an orienting reaction along with an increase in interest which precedes the Aha Insight (“Aesthetic Aha,” Muth and Carbon, 2013).

Based on our findings we would like to postulate a preliminary model of physical and semantical dynamics in the perception of indeterminate material and its effects on liking and interest (see Figure 13): an increase in complexity might signal the potential meaningfulness of a situation or object which makes this case more interesting. Such a tag on interest triggers an orienting reaction which unleashes further cognitive resources to process the potentially relevant item. By at least partly solving indeterminacy, a Gestalt is recognized which allows an Aha-insight to occur. The result of such an “Aesthetic Aha” is an increase in liking (as in Muth and Carbon, 2013).

## Conclusion

Perception and appreciation are evidently dynamic—and thus should be investigated by means of measures capturing such dynamics. This not only holds for dynamic stimulus material but also for the perception of a static object as it includes physical changes (for instance adapting one's own posture) and potential changes in semantics and appreciation over time and elaboration. Our results point to the importance of systematic analyses of such dynamics in art perception and appreciation by grasping the continuous nature of such experiences. We hope our preliminary model of dynamics in liking and interest, and their relation to complexity and perceptual insight inspires further research on the dynamics of perception and appreciation.

### Conflict of interest statement

The authors declare that the research was conducted in the absence of any commercial or financial relationships that could be construed as a potential conflict of interest.

## Supplementary material

**Supplementary Material A. The movie utilized as stimulus material can be watched here: http://vimeo.com/46138003**.

**Supplementary Material B. Impressions regarding the exhibition at the “Griesbadgalerie” can be gained here: http://vimeo.com/68991518**.

**Supplementary Material C. A visualization of the development of moments of insight can be found here: https://janus.allgpsych.uni-bamberg.de/CEP_Revision/insightlow.html**.
